# The Use of Adrenalin as an Addition to Solutions for Local Anesthesia

**Published:** 1902-11-15

**Authors:** Charles A. Elsberg


### THE USE OF ADRENALIN AS AN ADDITION TO SOLUTIONS FOR LOCAL ANESTHESIA.

BY CHARLES A. ELSBERG, M.D.

If one drop of the 1 : 1,000 solution of adrenalin chlorid be injected under the normal skin a slight burning sensation is felt, but no anesthesia occurs. Within one minute an area of skin, about two inches in diameter, becomes blanched and almost bloodless, and remains so from six to twelve hours. The same result will be achieved if a 1 : 5,000, or a 1 : 10,000, or a 1 : 15,000 solution be used; but with these weaker solutions the blanching of the tissues occurs only after a few minutes, and disappears only after three to six hours. After the blanching of the skin has disappeared the tissues apparently return to their normal condition.

The injections of adrenalin solutions were made under the normal skin of the forearm a considerable number of times, but no deleterious effects, such as sloughing or secondary ecchymoses, were ever observed. In the course of these investigations eucain and cocain solutions containing adrenalin chlorid, in the proportions of 1 : 5,000 to 1 : 20,000, were injected under the skin of the forearm. It was found that the anesthetic properties of the eucain and the cocain were perfectly preserved, while the adrenalin caused the same anemia and blanching of the tissues that had been observed after the injection of the pure adrenalin solutions. The blanching of the tissues extended from one to two inches beyond the area infiltrated, and continued for the same number of hours as when the plain adrenalin chlorid solutions were used.

During the past four months I have made a practice of adding adrenalin chlorid, in the proportion of 1 : 5,000 to 1 : 20,000, to solutions of eucain or cocain used for local anesthesia. I have employed these solutions in a number of minor operations, such as incision of abscesses and furuncles, the excision of sebaceous cysts, naevi, enlarged lymphatic glands, and the like. The amount of bleeding that occurred was remarkably small. It was even so slight that it was unnecessary to sponge off the wound a single time.

Only the larger vessels bled when cut across; the smaller ones are so tightly constricted by the adrenalin that no blood can escape from them, and therefore no oozing occurs. In none of the patients operated upon did even the smallest secondary hemorrhage occur, nor was any bleeding observed under the skin or at the site of the operation, when the first dressings were removed. In more than thirty clean operations primary union was in no manner interfered with or delayed.

The experiences recorded above have convinced me that the addition of adrenalin chlorid, in the proportion 1 : 5,000 to 1 : 20,000, to cocain solutions has a distinct value in minor operative surgery, in that it almost entirely does away with the oozing of blood from the wound. As adrenalin is a cardiac stimulant, it has the additional advantage that it will counteract the depressing effect of eucain or cocain. Since it keeps the local bloodvessels firmly contracted for a number of hours, it will prevent congestion, and hence the pain which is so apt to occour soon after the anesthetic effects of eucain or cocain have worn off.

Adrenalin chlorid solution can be obtained in a sterile condition of a strength of 1 : 1,000. The required amount of this solution should be added to a sterile solution of eucain, cocain, or Schleich's mixture, just before it is to be used. The best results can be obtained with solutions which contain 1 : 5,000 to 1 : 10,000 parts of adrenalin chlorid. If it be necessary to use a large quantity, it will be advisable to select a 1 : 15,000, or even 1 : 20,000 solution.—*American Medicine.*

